# Draft Genome Sequence of “*Candidatus* Spirobacillus cienkowskii,” a Pathogen of Freshwater *Daphnia* Species, Reconstructed from Hemolymph Metagenomic Reads

**DOI:** 10.1128/MRA.01175-18

**Published:** 2018-12-06

**Authors:** Luana Bresciani, Leandro N. Lemos, Nina Wale, Jonathan Y. Lin, Alexander T. Strauss, Meghan A. Duffy, Jorge L. M. Rodrigues

**Affiliations:** a Soil Microbiology Laboratory, Department of Soil Science, Luiz de Queiroz College of Agriculture, University of São Paulo, Piracicaba, São Paulo, Brazil; b Cell and Molecular Biology Laboratory, Center for Nuclear Energy in Agriculture, University of São Paulo, Piracicaba, São Paulo, Brazil; c Department of Ecology and Evolutionary Biology, University of Michigan, Ann Arbor, Michigan, USA; d Department of Land, Air, and Water Resources, University of California, Davis, California, USA; e Department of Biology, Indiana University, Bloomington, Indiana, USA; f Environmental Genomics and Systems Biology Division, Lawrence Berkeley National Laboratory, Berkeley, California, USA; University of California, Riverside

## Abstract

We report here the near-complete genome sequence of “*Candidatus* Spirobacillus cienkowskii,” a spiral-shaped, red-pigmented uncultivated bacterial pathogen of *Daphnia* spp. The genome is 2.74 Mbp in size, has a GC content of 32.1%, and contains genes associated with bacterial motility and the production of carotenoids, which could explain the distinctive red color of hosts infected with this pathogen.

## ANNOUNCEMENT

The species “*Candidatus* Spirobacillus cienkowskii” is a deep-branching uncultivated Deltaproteobacteria pathogen of freshwater daphniids, which are important members of aquatic food webs (1). The most distinctive phenotypic characteristic associated with its infection is the red color in the host hemolymph (2). We used high-throughput sequencing and computational binning approaches to assemble and reconstruct the pathogen genome, previously described only through sequences of 16S rRNA and DNA primase β-subunit (*gyrB*) genes (3).

Daphnia dentifera organisms infected with “*Candidatus* Spirobacillus cienkowskii” were originally collected from Dogwood Lake (38°32′37ʺN, 87°03′04ʺW; Greene-Sullivan State Forest, IN). Infections were propagated in D. dentifera L6D9 and the “standard” genotype collected from Dogwood Lake and a lake in Barry County, Michigan, respectively. The hemolymph from 43 infected hosts was collected and pooled for DNA extraction using the QIAamp DNA minikit (Qiagen, Germantown, MD) following manufacturer instructions. Metagenomic reads were generated with the Illumina MiSeq platform using paired-end 350-bp sequencing, and a total of 3,257,849 paired-end reads were obtained. Low-quality reads (<100 bp and a Phred score of <30) were filtered using Cutadapt v.1.18 (4), and the genome was assembled using IDBA-UD v.1.1.1 (5). A binning strategy was used to reconstruct the genome by taking into consideration the GC content and coverage of clustering contigs into individual genome populations through MaxBin v.2.0 (6) and manual curation with CheckM v.1.0.5 (7). To validate the genome reconstruction, BLASTN v.2.7.1+ (8) was used to compare nucleotide coding sequences (CDSs) to sequences of “*Candidatus* Spirobacillus cienkowskii” 16S rRNA (GenBank accession number EU220836) and *gyrB* (EU220837) genes deposited in GenBank.

Functional annotation was performed using PATRIC v.3.5.25 (9), Prokka v.1.12 (10), and KAAS v.1.0 (11). The “*Candidatus* Spirobacillus cienkowskii” genome was assembled into 126 contigs (2,739,001 bp with a GC content of 32.1%) with an *N*_50_ value of 39,228 bp. Quality control of the genome assembly indicated a near-complete genome (91.2% completeness) without contamination (0%). Comparative analysis with available 16S rRNA and *gyrB* genes for “*Candidatus* Spirobacillus cienkowskii” showed 100% identities with our genome bin. A total of 2,553 CDSs, 37 tRNAs, and 1 complete rRNA operon (23S, 16S, and 5S rRNA) were detected in the genome. Of the total number of proteins, 37% had functional assignments (961 proteins), 15% had gene ontology assignments (402), and 46% had FIGfam assignments (1,176). Several genes associated with phytoene production (terpenoid backbone biosynthesis), the colorless precursor of all C_40_ carotenoids (12), and other genes associated with carotenoid synthesis were detected (13). We identified genes associated with flagellar biosynthesis and assembly, which may be used for movement into the host hemolymph or for facilitating transmission to a new host.

*Daphnia* spp. are key members of lake food webs (14), and pathogen outbreaks reduce the host population growth rate and density and elevate the death rate (15). The “*Candidatus* Spirobacillus cienkowskii” genome will increase our knowledge of host-pathogen interactions. The annotated genome will help microbiologists identify conditions for isolating this ecologically important but as yet uncultivated pathogen.

### Data availability.

The whole-genome shotgun project of “*Candidatus* Spirobacillus cienkowskii” has been deposited at DDBJ/ENA/GenBank under the accession number QOVW00000000. The version described in this paper is version QOVW01000000. The raw reads were deposited in the Sequence Read Archive (SRA) under the accession number PRJNA450308.
